# Effectiveness of flipped classroom in pharmacy education – a meta-analysis

**DOI:** 10.1186/s12909-023-04865-2

**Published:** 2023-11-17

**Authors:** He Cui, Xinyu Xie, Boyang Wang, Yuan Zhao

**Affiliations:** 1 Hebei GEO University, Shijiazhuang, Hebei Province 050000 PR China; 2 Department of Orthodontics, Shijiazhuang Second Hospital, Shijiazhuang, 050000 PR China; 3 Hebei Academy of Education Sciences, Hebei Education Department, Shijiazhuang, 050000 PR China; 4 Hebei Vocational University of Industry and Technology, Shijiazhuang, 050000 PR China

**Keywords:** Flipped classroom, Meta-analysis, Pharmacy education, Flipped learning, Learning performance

## Abstract

**Background:**

Flipped classroom, blended with online and offline learning, was regarded as an effective learning approach in pharmacy education. This meta-analysis was to comprehensively compare the effectiveness of flipped classroom and traditional lecture-based approaches, attempting to generate a unified and firm conclusion of the effectiveness of flipped classroom in pharmacy education.

**Methods:**

Data were collected from 7 databases, involving Cochrane Library, PubMed, Embase, ScienceDirect, Web of Science, China National Knowledge Infrastructure (CNKI), and Chinese Biomedical Literature Service System (SinoMed). The studies were included if they included objective evaluation of students’ performance between groups of flipped classroom and traditional approaches. The standardized mean difference (SMD) with a corresponding 95% confidence interval (95% CI) was used as the outcomes for data pooling.

**Results:**

A total of 22 studies (28 comparing groups) with 4379 participants were included in the meta-analysis. The risk of bias was relatively high. Results of the analysis revealed that flipped classroom presented significant advantages over traditional approaches in student performance improvement, with no evidence of publication bias. Through subgroup analysis, it showed better outcomes for flipped classrooms over traditional lectures for the other subgroups, including different performance, degree programs.

**Conclusions:**

Current evidence reveals that the flipped classroom approach in pharmacy education yields a statistical improvement in student learning compared with traditional methods. In the future, instructors should introduce more online technology into classroom and construct an interactive learning environment.

**Supplementary Information:**

The online version contains supplementary material available at 10.1186/s12909-023-04865-2.

## Introduction

Flipped classroom, a form of blended learning, took advantage of asynchronous lectures and in-class interactive activities. Flipped classroom is defined as a method that instructors expose pre-work to students outside of class, and then use class time to arrange the harder work of helping assimilate that knowledge, through problem-solving, discussion, or debates [1].

One of the most important advantages of flipped classroom is student-centered learning, through which students can actively engage in classroom and interact more with instructors [2]. Before class, students have already familiarized the learning contents through posted materials. Then, lecturers undertake a series of activities to inspire the interests of students, including presentations, patient case discussions, classroom games. These interactive learning activities transform passive acceptance into active learning, thus enhancing critical thinking and innovation ability [2]. Additionally, through group discussions and problem-solving processes, students are able to learn more effectively from their fellow students rather than instructors [3, 4].

Recent years have witnessed the increasing application of flipped classrooms in various fields of health professions education, including pharmacy education [5–7]. Additionally, several systematic reviews have been undertaken in evaluating its effectiveness of it on pharmacy education: K. S. Chen et al. conducted a meta-analysis to examine the efficacy of flipped classroom in health professions based on effect measures(examination scores, course grades, OSCEs) [5]. They not only incorporated controlled studies between the experimental group and the control group, but also included before-and-after studies. The meta-analysis included a subgroup analysis on pharmacy education, which showed an advantage of the flipped classroom for both examination grade(n = 7, 95%confidence interval SMD 0.53 [0.12,0.93]) and course grade (n = 3, 95%confidence interval SMD 0.53 [0.35,0.71]). They both have a high degree of statistical heterogeneity. Hew & Lo studied the effects of flipped classroom on health education, including subgroup analysis of pharmacy education [7]. The results also favored flipped classroom (n = 10, 95% confidence interval SMD 0.45 [0.24,0.65]), which has a high degree of statistical heterogeneity. Gillette et al. compared outcomes between a traditional and flipped classroom in pharmacy schools (n = 6, 95% confidence interval WMD 2.90 [-0.02,5.81]). Because of limited trials, it did not conduct subgroup analysis [6]. Besides, it included trials with different lecture hours between experimental and controlled groups.

These three studies included different trials and lacked subgroups to analyze effectiveness from different perspectives toward pharmacy education. Therefore, we undertook this meta-analysis to systematically evaluate the effectiveness of flipped classrooms in pharmacy education. We attempted to include more trials on the effects of flipped classroom towards pharmacy students, to generate a unified and firm conclusion regarding its efficacy. In addition, subgroup analysis was conducted to assess the impact of factors on the effectiveness of flipped classroom.

## Methods

This meta-analysis was conducted under the Preferred Reporting Items for Systematic Reviews and Meta-analysis (PRISMA) guidelines. The protocol was registered in the International Platform of Registered Systematic Review and Meta-analysis Protocols (INPLASY). The registration number is INPLASY202380130. Available from: https://inplasy.com/inplasy-2023-8-0130/.

### Search strategy

Data were collected up to October 10th, 2022 from the following databases: Cochrane Library, PubMed, Embase, ScienceDirect, Web of Science, China National Knowledge Infrastructure (CNKI), and Chinese Biomedical Literature Service System (SinoMed). The following keywords were selected: ((flipped classroom) OR (flipped education) OR (flipped learning) OR (reverse classroom) OR (backward classroom) OR (inverted classroom) OR (inverse classroom)) AND (pharmac*). The search strategy was imported as a string and searched independently in these 7 databases.

### Inclusion and exclusion criteria

Study design: We included studies designed to explore the effectiveness of flipped classroom in pharmacy education in comparison with traditional classroom or lecture-based pedagogy. The studies should include objective evaluation of students’ performance, like course grades or GPA. There are three types of study design included, including randomized controlled trial(RCT), quasi-experiment, and historical(retrospective) cohort study. For RCT, the students are randomly distributed into groups in both control and experimental groups, which was adopted by traditional face-to-face classroom and flipped classroom separately. Quasi-experiment is a trial in which the subjects are not randomly distributed into groups. For example, flipped classroom and traditional face-to-face classroom was adopted for two different classes, which was not randomly distributed. In a historical cohort control study, researchers conduct flipped classroom and compare the student performance of flipped classroom with preexisting historical data of traditional classroom, like test scores in previous years. Before–and-after study was excluded from the meta-analysis. For before-and-after studies, researchers only compare student performance before and after adopting flipped classrooms.

*Participants*: Pharmacy students, who attended courses in pharmacy curriculum from higher education programs, were included in this meta-analysis.

*Intervention*: Flipped classroom was conducted in experimental groups, which included pre-work prepared by teachers, self-directed learning before class, and in-class interactive activities between students and lecturers, while control groups were equipped with a traditional lecturer-centered teaching method as a comparison. The course should ensure the same credit hours, class time, and same course topics prepared for students between the experimental and control groups.

*Outcomes*: Course grades or examination scores served as main indicators to evaluate the effects of the flipped classroom and traditional lectures. The contents and forms of these assessment instruments must be similar or identical between the experimental and control groups.

*Exclusion criteria*: Articles were excluded if: published studies lacked the required control group; published studies lacked sufficient extractable data or calculable effect size; students included in the meta-analysis were from K-12 education; written language was not English or Chinese; studies were published before 2000.

### Data extraction method

Two authors independently reviewed each article, and extracted data involving the first author, published year, countries, sample size, pharmacy course type, student level, intervention measures, contrast pedagogy, and outcome indicators. When there were different opinions, the authors resolved them through discussion or adjudication by the third reviewer.

### Assessment of methodological quality

The Effective Public Health Practice Project (EPHPP) Quality Assessment Tool was employed to assess the methodological quality of studies, due to its suitability for both interventional and observational studies [8]. According to the EPHPP tool, the following items were taken into consideration: selection bias; study design; confounding factors; study blinding; data collection; withdrawals and dropouts. The quality of studies was rated as Strong, Moderate, and Weak. Based on the number of weak ratings they received, the overall rating was also rated as three levels: Strong (no weak ratings), Moderate (one weak rating), and Weak (two or more weak ratings).

### Statistical analysis

Quantitative analysis was undertaken by the Stata/SE version 16 (StataCorp LLC, College Station, TX). The standard mean difference (SMD) with the random-effects model was adopted for data pooling, which extracts average mean and standard deviations (SDs) from studies. Considering that educational research usually included multiple effect measures, we extracted the data separately according to the types of effect measures. When a study incorporated several similar effect measures, we chose the most suitable one to present the outcome required for the meta-analysis. If one study included different students’ performance data used to evaluate different parts or modules of one course, we incorporated these independent group comparisons separately. For instance, subsets of examination were usually used to evaluate different modules of a course, for precisely evaluating student performance. For studies that lacks of required data, such as average means and SDs, we contacted the authors by email. Studies would be excluded if we could not obtain the required data. I-squared statistics were conducted to evaluate the heterogeneity of effect sizes. An I2 of < 25% represents low heterogeneity, 25-50% moderate heterogeneity, and > 50% high heterogeneity. If it has shown a high heterogeneity, subgroup analysis would be performed to evaluate source of the heterogeneity, including outcome measures, research design, degree programs, countries, format of tests, etc. Begg’s test and Egger’s regression test were used to assess the publication bias quantitively, while visual analysis from the funnel plot was explored simultaneously.

## Results

### Literature search results

**Fig. 1:**
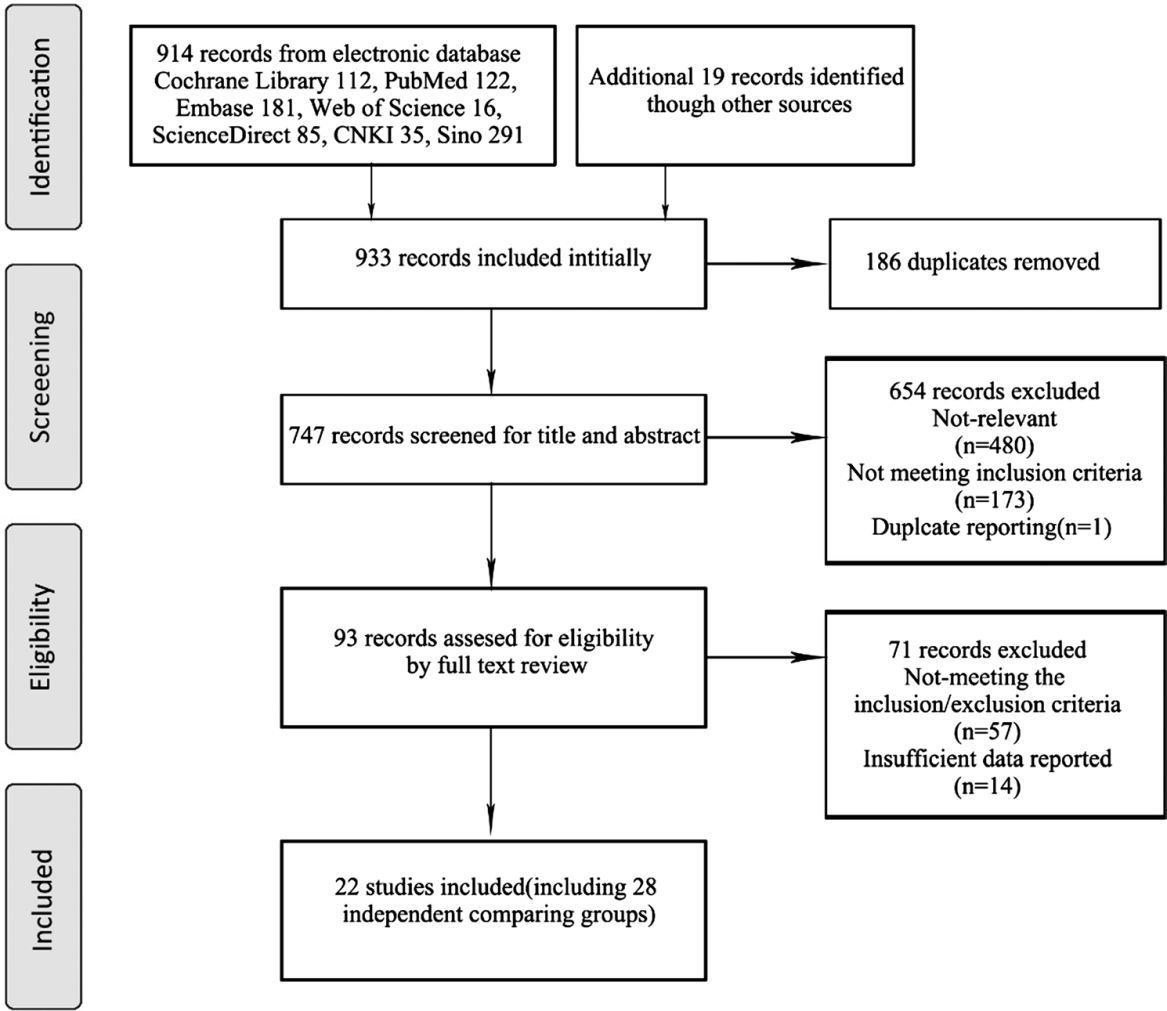
Flow-process diagram for the study selection and inclusion. CNKI, China National Knowledge Infrastructure; SinoMed, Chinese Biomedical Literature Service System

A PRISMA flow diagram represents the search process (see Fig. 1). There are 933 records retrieved from data pooling, in which 914 records were searched from seven datasets, and 19 studies were added through references. 911 studies were removed in the process of selection, of which 186 studies were screened for duplication, 654 studies were filtered for viewing title and abstract, and 71 studies were removed by full-text screening. Finally, the remaining 22 studies (including 28 comparing groups) with 4379 participants, were included in the meta-analysis [2, 9–29]. Among these studies, Prescott et al. included 2 comparing groups, Wong et al. contained 3, while Sumanasekera et al. included 4.

### Characteristics of included studies

The publication year of all 22 articles covering the period between 2014 and 2022, with sample sizes ranging from 35 to 588 pharmacy students in participation (see Table 1 for details). Of the 22 included studies, 21 were journal articles and 1 was conference abstract. In terms of design, they included 1 RCT, 3 quasi-experimental studies, and 18 historical control studies. Among these studies, 16 studies originated from the USA, 3 from China, 1 from Thailand, 1 from Malaysia, and 1 from Qatar. For outcome measures, there are 2 adopted course grades, 18 used exam scores, 1 equipped with OSCE, and one study included both final exam scores and OSCE. Among the 18 studies that adopted exam scores, 11 employed MCQs (multiple choice questions) in exams, 1 used calculation questions, 1 equipped with fill-in-blank and short answer questions, and 6 adopted blended test formats. For degree programs students attended, there are 8 studies for Doctor of Pharmacy (PharmD), 2 for Undergraduate program, 1 for Graduate program, and 1 for an Associate degree program.

**Table 1:** Characteristic of included studies

NO.	Study ID		Sample		Study design	Country of Origin	Source	Participants (level/course)	Format of outcome
			CG	IG					
1	Prescott, et al. 2016	comp1	108	123	Historical control	US	Journal	1st year PharmD/Patient Assessment 1	Course grade
		comp2	97	129	Historical control	US	Journal	2nd year PharmD/Patient Assessment 2	Course grade
2	He, et al. 2019		56	81	RCT	CN	Journal	Undergraduate/Pharmaceutical Marketing and Management	Exam score (5 short open-ended questions, 10MCQs and 2 essays)
3	Kangwantas, et al. 2017		21	29	Historical control	Thailand	Journal	2nd year pharmD/Fundamental Nutrition	Final exam scores (MCQs)
4	Gloudeman, et al. 2018		104	102	Historical control	US	Journal	1st year pharmacy/Pharmaceutical Calculation	Exam scores (13 calculation exam questions)
5	Goh, et al. 2019		74	63	Historical control	Malaysia	Journal	2nd year pharmacy/Dosage Form	Exam scores (5 MCQs and 2 essay)
6	Taglieri, et al. 2017		283	305	Historical control	US	Journal	3rd year PharmD/Over-the-Counter Drugs, Self-Care Products	Course grade
7	Lockman, et al. 2017		156	162	Historical control	US	Journal	1st year PharmD/Pharmacology and Therapeutics	OSCE AND Final exam scores (MCQs)
8	Koo, et al. 2016		89	89	Historical control	US	Journal	2nd year PharmD/Integrated Pharmacotherapy	Final exam scores (MCQs)
9	Munson, et al. 2015		125	113	Historical control	US	Journal	1st professional year pharmacy/Essentials of Pharmacogenomics	Final exam (10 blended questions)
10	Wong, et al. 2014	comp1	105	101	Historical control	US	Journal	1st year pharmacy/Cardiac Arrhythmias (basic sciences)	Final exam scores (MCQs)
		comp2	105	101	Historical control	US	Journal	1st year pharmacy/Cardiac Arrhythmias(pharmacology)	Final exam scores (MCQs)
		comp3	105	101	Historical control	US	Journal	1st year pharmacy/Cardiac Arrhythmias(therapeutics)	Final exam scores (MCQs)
11	Bossaer, et al. 2016		72	76	Historical control	US	Journal	3rd professional year/Pharmacotherapy	Final exam score (15 matching questions, 60 MCQs)
12	Cotta, et al. 2016		165	151	Historical control	US	Journal	1st year pharmacy students/Pharmaceutical Calculations	Final exam scores (Short answer or fill-in-blanks)
13	McLaughlin, et al. 2014		153	162	Historical control	US	Journal	1st year PharmD/Basic Pharmaceutics	Final exam scores (MCQs)
14	McLaughlin, et al. 2013		13	22	Historical control	US	Journal	1st year pharmacy/Basic Pharmaceutics II	Final exam scores (MCQs)
15	Pierce, et al. 2012		68	71	Historical control	US	Journal	Undergraduate and Graduate/Renal Pharmacotherapy Module	Final exam scores (MCQs)
16	Stewart, et al. 2013		65	71	Historical control	US	Journal	PharmD/Pharmacotherapy Course Module	Final exam scores (MCQs)
17	Donihi, et al. 2014		123	133	Historical control	US	Conference	2nd year PharmD/Gastroenterology, Nutrition	Final exam scores (MCQs)
18	Sumanasekera, et al. 2020	comp1	91	73	Historical control	US	Journal	2nd year pharmacy/Pharmacology and Medicinal Chemistry-Hypertension	Final exam scores (8 to 12 MCQs)
		comp2	91	73	Historical control	US	Journal	2nd year pharmacy/Pharmacology and Medicinal Chemistry-Kidney and Diuretics	Final exam scores (8 to 12 MCQs)
		comp3	91	73	Historical control	US	Journal	2nd year pharmacy/Pharmacology and Medicinal Chemistry-Diabetes	Final exam scores (8 to 12 MCQs)
		comp4	91	73	Historical control	US	Journal	2nd year pharmacy/Pharmacology and Medicinal Chemistry-Cardiovascular	Final exam scores (8 to 12 MCQs)
19	Nazar, et al. 2019		63	69	Historical control	Qatar	Journal	Master of Pharmacy/Pharmacy Law	Final exam scores (MCQs)
20	Anderson, et al. 2017		32	38	Quasi -experiment	US	Journal	1st year pharmacy /Pharmaceutical Calculations	OSCE
21	Chen, et al. 2020		44	49	Quasi -experiment	CN	Journal	Associate program/Medicinal Botany	Final exam scores
22	Wang, et al. 2019		30	30	Quasi -experiment	CN	Journal	Undergraduate/Individualized Medication of Cardiovascular Drugs	Final exam scores

CG, Controlled groups; IG, Intervention groups; CN, China; MCQ, Multiple choice questions; OSCE: Objective Structured Clinical Examination

### Quality assessment

We assessed the methodological quality of 22 incorporated studies, among which 19 studies exhibited a Strong risk of bias, and 3 studies with Moderate risk of bias (see Table 2 for details). According to assessing results, the Strong risk of bias mainly came from blinding, data collection, confounders, and withdrawals/drop-outs judgment domains. Specifically, the majority of the studies did not present important quality indicators, leading to poor reporting quality and a high risk of bias. The missing information included the eligible population, dropout populations, characteristics of participants, important confounders between groups, and the reliability/validity of assessment tools.

**Table 2:** Quality assessment of included studies

NO.	Study ID	EPHPP components						Overall rating
		Selection bias	Study design	Confouders	Blinding	Data collection	Withdrawals/droupouts	
1	Prescott, et al. 2016	2	2	3	3	2	3	3
2	He, et al. 2019	1	1	1	3	2	1	1
3	Kangwantas, et al. 2017	2	2	3	3	3	3	3
4	Gloudeman, et al. 2018	2	2	3	3	3	3	3
5	Goh, et al. 2019	2	2	2	3	3	2	3
6	Taglieri, et al. 2017	2	2	3	3	2	3	3
7	Lockman, et al. 2017	2	2	1	3	2	1	2
8	Koo, et al. 2016	2	2	1	3	3	1	2
9	Munson, et al. 2015	2	2	3	3	3	3	3
10	Wong, et al. 2014	2	2	1	3	3	1	2
11	Bossaer, et al. 2016	3	2	3	3	3	3	3
12	Cotta, et al. 2016	2	2	3	3	3	3	3
13	McLaughlin, et al. 2014	2	2	1	3	2	3	2
14	McLaughlin, et al. 2013	2	2	3	3	3	1	3
15	Pierce, et al. 2012	3	2	3	3	3	3	3
16	Stewart, et al. 2013	2	2	2	3	2	3	2
17	Donihi, et al. 2014	3	2	3	3	3	3	3
18	Sumanasekera, et al. 2020	3	2	3	3	3	3	3
19	Nazar, et al. 2019	2	2	3	3	3	3	3
20	Anderson, et al. 2017	1	1	1	3	2	1	1
21	Chen, et al. 2020	2	2	3	3	3	3	3
22	Wang, et al. 2019	2	1	3	3	3	1	3

EPHPP, The Effective Public Health Practice Project Quality Assessment Tool; 1 = Strong methodological quality;2 = Moderate methodological quality;3 = Poor methodological quality

### Data synthesis

**Fig. 2:**
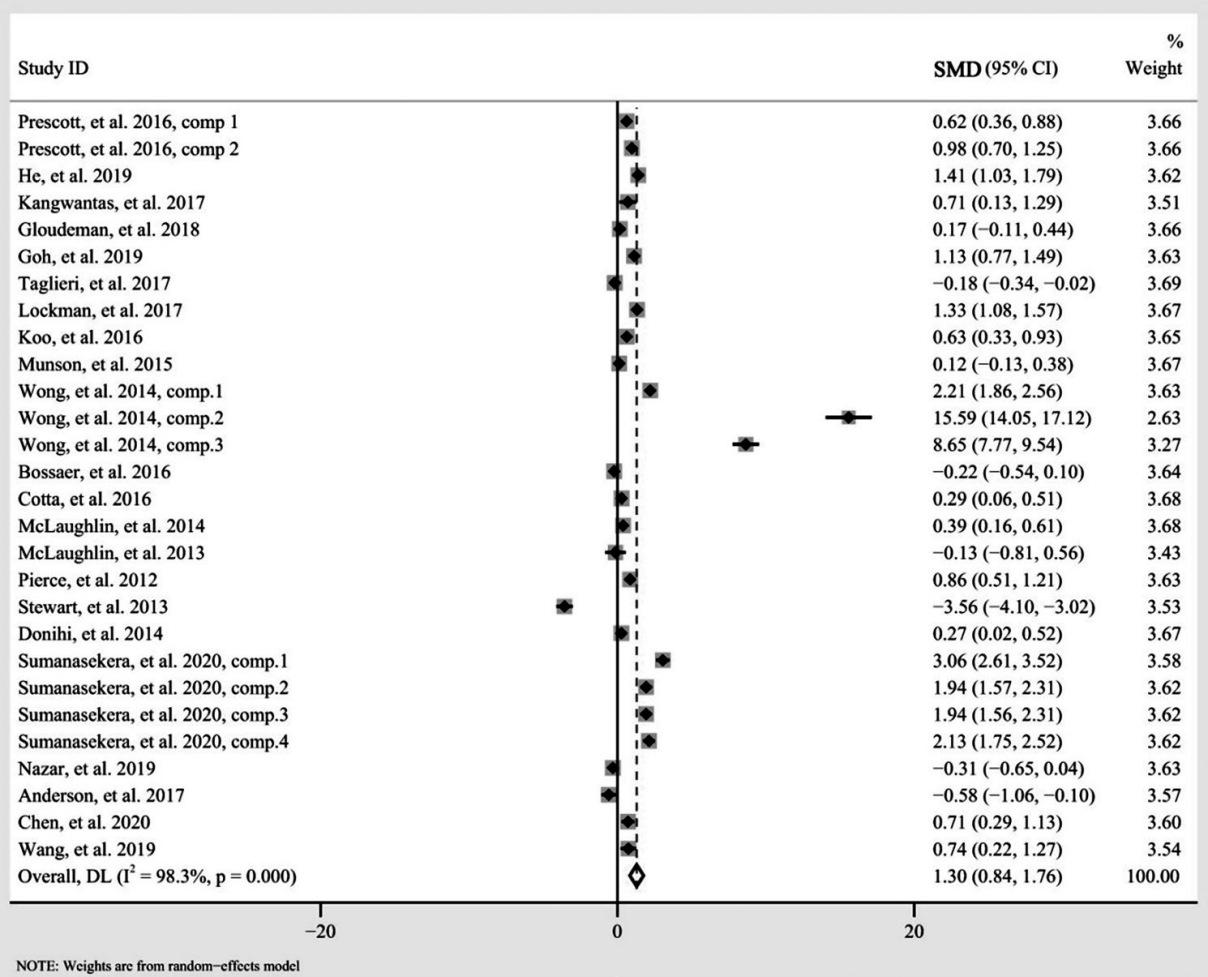
Forest plot for the effectiveness of flipped classroom vs. traditional lectures. SMD, Standardized Mean Difference; CI, Confidence Interval; comp, comparing groups

A total of 22 studies, including 28 comparing groups, provided a comparison of effects on pharmacy students’ performance between flipped classroom and traditional approaches. The random effect model was applied because of significant heterogeneity across studies (I^2^ = 98.3%). According to meta-analysis results (see Fig. 2), flipped classroom significantly promoted the pharmacy students’ performance compared with the lecture-based learning approach (SMD 1.30, 95%CI 0.84–1.76, P < 0.001).

### Subgroup meta-analysis

From different perspectives, subgroup meta-analyses were conducted to evaluate the effects of flipped classroom compared to traditional methods. The scope of subgroup analysis included all the subgroups more than 2 comparing groups. The results showed that there were still significant heterogeneities in different subgroups (see Table 3).

**Table 3:** Analysis results of subgroups

Subgroup and domain	n	Effects		Heterogeneity	
		SMD (95% CI)	p-value	I^2^,%	p-value
Outcome measures					
Exam scores	24	1.82 (1.71,1.93)	< 0.001	99.00%	< 0.001
Course grade	3	0.25 (0.13,0.38)	< 0.001	96.70%	< 0.001
OCSE	2	0.98 (0.77,1.20)	< 0.001	97.90%	< 0.001
Research design					
RCT	1	1.41 (1.03,1.79)			
Historical control	3	1.58 (1.49,1.68)	< 0.001	99.00%	< 0.001
Quasi-experiment	24	0.31 (0.04,0.58)	0.023	89.60%	< 0.001
Degree programs					
Doctor of Pharmacy	9	0.24 (0.15,0.32)	< 0.001	97.60%	< 0.001
Graduate	1	-0.31 (-0.65,0.04)			
Undergraduate	2	1.2 (0.9,1.51)	< 0.001	75.30%	0.044
Associate	1	0.71 (0.29,1.13)			
Country					
US	22	1.63 (1.53,1.72)	< 0.001	99.10%	< 0.001
Non-US	6	0.74 (0.58,0.91)	< 0.001	90.40%	< 0.001
Format of test					
MCQ	16	2.57 (2.41,2.73)	< 0.001	99.40%	< 0.001
Calculation questions	1	0.17 (-0.11,0.44)			
Short answer and fill-in-blanks	1	0.29 (0.06,0.51)			
Blend	6	0.62 (0.49,0.75)	< 0.001	88.50%	< 0.001
Course type					
Pharmacy course	26	1.58 (1.48,1.67)	< 0.001	99.00%	< 0.001
Interdisciplinary course	2	0.57 (0.31,0.82)	< 0.001	97.70%	< 0.001
Flipped classroom design					
Short recorded video pre-class (average duration < 18 min)	6	0.63 (0.37,0.88)	0.004	70.8	< 0.001
Quiz at start of in-class?	9	3.91 (3.66,4.16)	< 0.001	99.70%	< 0.001
Incorporating patient case discussion in-class?	18	2.14 (2.02,2.27)	< 0.001	99.30	< 0.001

SMD, Standardized Mean Difference; CI, Confidence Interval; OCSE, Objective Structured Clinical Examination; RCT, Randomized Controlled Trial; MCQ, Multiple Choice Questions

For outcome measures, it showed significant improvements for flipped classroom in exam scores, course grades, and Objective Structured Clinical Examination (OCSE) separately. Lockman et al. included both final exam scores and OCSE, which support the teaching effects of flipped classroom [21]. Based on the format of the test, subgroup analysis was conducted for the outcome of final examination scores. Significantly higher examination scores can be identified for both MCQ (multiple-choice-question) and blend test format for the flipped classroom condition.

For research design, historical control studies and quasi-experiments both revealed an advantage for flipped classroom over the traditional classroom.

The subgroup analysis by country of origin showed that the advantage of flipped classroom was observed in both US and Non-US countries. Non-US studies were all from Asia, involving China, Qatar, Thailand, and Malaysia.

For degree programs, an advantage of the flipped classroom over the traditional classroom condition was present in both PharmD and Undergraduate programs. Studies with PharmD programs were from the US and Thailand, while the Undergraduate programs were all from China.

In order to evaluate the pedagogic design of the flipped classroom, four subgroups were separated for meta-analysis, including “incorporating patient case scenario”, “interdisciplinary courses”, “pre-class video less than 18 mins”, and “availability of quiz at the start of class”.

It is reported that flipped classroom have advantages in problem-solving, so flipped classroom incorporating patient case scenario was analyzed. There were 18 comparing groups, showing a better effect of flipped classroom model (SMD 2.14, 95%CI 2.02, 2,27).

There were two interdisciplinary courses, one of which was a study in Pharmacy and Marketing by He et al., while the other is Pharmacy and Law by Nazar et al. Better effects can also be observed in the analysis results of these two studies (SMD 0.57, 95% CI 0.31, 0.82).

There were 7 comparing groups included to investigate the effectiveness of “pre-class video less than 20 mins”. It proved the subgroup had a better effect in flipped classroom over traditional classrooms (SMD 0.32, 95%CI 0.22, 0.42).

The subgroup of “availability of quiz at the start of class”, incorporating 9 comparing groups, showed an advantage for flipped classroom (SMD 3.91, 95%CI 3.66, 4.16).

### Publication bias

A visual analysis through the funnel plot revealed obvious evidence of asymmetry, which was regarded as significant publication bias. In addition, the result is further confirmed by Egger’s regression test (0.007) and Begg’s test (0.028), whose P values were both lower than 0.05.

## Discussion

Through the results of meta-analysis, flipped classroom applied in pharmacy education course has advantages over the traditional lecture-based approach in increasing students’ learning performance.

Flipped classroom provides practical activities in the classroom, which pharmacy higher education lacks [30, 31]. Flipped classroom can help pharmacy education keep up with the information age, which incorporates online learning tools and quick techniques to help students to be involved in practical tasks [32]. Additionally, flipped classrooms incorporate a series of interactive activities, including problem-based scenario discussion, through which students could promote their operational skills and better adapt to the developing pharmacy profession.

Flipped classroom provides short videos to promote engagement and hold the attention of pharmacy studnets. It is reported that medical students can only keep their focus for 15 to 20 min at the beginning of class [2]. Student attention would decline in the classroom since their brain has limited attention span capabilities, especially for pharmacy students who need more memorization [33]. Take TED Talks for example, these influential videos from expert speakers are limited to 18 min, which is long enough to present basic content and short enough to hold a person’s attention [33]. Therefore, when instructors prepared videos for flipped classroom, they intentionally recorded short videos or divided recordings of the entire lecture into mini-lectures. Based on the experience from TED, the duration of videos lower than 18 min were selected for subgroup analysis. Through evaluating on 6 trials of pharmacy education, it reflected an advantage of flipped classroom with short videos over traditional approach.

Flipped classroom allows pharmacy students to work with different majors. When adopting flipped classroom on interdisciplinary courses, students from different disciplines could learn from each other through interactive activities and team-based discussions [25, 26]. Therefore, when pharmacy students graduate from university to work, they would easily adjust to collaboration with other professionals and practice on interdisciplinary teams [34]. Since the flipped classroom is good at arranging interactive activities through divided teams, we estimated that flipped classroom has advantages in promoting student performance in interdisciplinary courses. Through data extraction, there were only two comparing groups included in our subgroup meta-analysis, which showed an advantage over the traditional method with high heterogeneity. It still needs further study with more included comparing groups to get a firm conclusion.

Flipped classroom model can enhance the long term retention of information, especially for pharmacy students who requires a lot of memorization and recitation [35, 36]. It includes several retrieval processes: After watching pre-class videos, instructors assign questions to recall key concepts in videos; at the start of class, students receive quizzes developed by instructors; in addition to monthly exams and final exams, repeatedly retrieval of information helps students master knowledge firmly, and a better learning performance accordingly. Khe Foon HEW and Chung Kwan LO stated that the flipped classroom would be more effective when instructors use quizzes at the start of in-class sessions [7]. In our meta-analysis, quizzes at the start of the class were also evaluated, reflecting a better effect of flipped classroom.

### Strength and limitations

The results of meta-analyses have several limitations. First, the studies had a high degree of statistical heterogeneity, which does not make it possible to make firm conclusions. High heterogeneity mainly results from the diversity of flipped classroom format. Instructors have a different understanding of flipped classroom, so the actual implementation varies significantly, thus influencing actual learning effects. In addition, there exists significant publication bias. To avoid missing important information and publication bias, we incorporated conference studies and journals from different countries, which might lead to uneven methodological qualities. Third, we only included studies in English and Chinese for meta-analysis, while trials in other languages that may met the criteria were excluded. These missing studies might lead to heterogeneity in the analysis.

Even though there are several limitations, our study has more strengths. Our overall findings are built based on three related meta-analysis results before. After comparing their included studies with ours, several of them had been excluded from our studies. Some excluded studies cannot meet our inclusion criteria, while others miss necessary data, even though we contacted the original authors by email. Finally, we included 22 articles with 28 comparing groups, involving 4379 participants in our meta-analysis. As far as we know, this is the largest meta-analysis to evaluate the effectiveness of flipped classroom vs. traditional classroom for pharmacy students, which offers a higher level of evidence. In addition, we separated subgroups from included studies based on a series of factors, involving research method, degree program, flipped classroom format, and so on. According to the differences in results, we have a deeper understanding of nuances within flipped classroom methods, compared to evaluating the data as a whole group.

### Further research

The blended learning approach represented by flipped classroom, not only absorbed the advantages of online learning and face-to-face traditional methods, but also encouraged active learning rather than simply transmitting the information [37]. When adopting flipped classroom, instructors should employ more interactive activities to strengthen the retrieval learning process. For example, Sumanasekera et al. introduced crossword puzzle games and Kahoot web-based interactive gaming; Wong et al. arranged active learning exercises based on characteristics of different modules, which includes reading and interpreting electrocardiograms in basic sciences, while equipping performing calculations for pharmacology, cardiac arrhythmia patient cases discussion and management for therapeutics module [13, 29]. Instructors need to continually make innovations and add various retrieval practices in the flipped classroom, improving student performance by long detention of learning.

Additionally, instructors should take full advantage of flipped classroom to promote students’ practical skills. With the development of the pharmacy profession, the role of pharmacists is transferring from previous dispensing role to a more patient-focused and outcome-oriented role [30]. Several of our included studies have introduced patient-based scenarios and encouraged real case discussion. For example, Pierce et al. encouraged student engagement in the patient case problem-solving process, including making interventions, patient assessment, drug dosing in therapy, and pharmacokinetic calculations [38]. Therefore, instructors need to find more suitable patient cases and guide students to engage in discussion and problem-solving. With patient-based scenarios, flipped classroom can link up with pharmacy education and profession, preparing students for practice in pharmacy.

What’s more, according to student perception, there is a universe comment that they have more time commitments than traditional methods, especially for the pre-class time. Gloudeman et al. reported that parts of pharmacy students even spend more than 3 h preparing flipped classroom [23]. He et al. studied the time investment in the learning of flipped classroom and the traditional approach, in which students spent more time in pre-class and less time after class [25]. There is no doubt that the time commitment can make a difference in leaning performance, but the current study missed the detailed learning time. For further study, instructors may record total learning time and study the relationship between the time and student performance.

Besides, more detailed records are needed for further meta-analysis. When extracting data for analysis, several important pieces of information was not described clearly in studies, including test format, video durations, and deviations. Therefore, many of them were excluded from our analysis or subgroup analysis. There are only 22 studies included in the meta-analysis, and even less studies when adopting subgroup analysis, which might lead to a high degree of statistical heterogeneity. For further study, researchers should record more detailed information for next trial, and include more studies in the next meta-analysis, which not only contribute to a comprehensive understanding of flipped classroom, but also reduce risks of bias and lead to a firmer conclusion.

## Conclusion

The results of the meta-analysis revealed that flipped classroom approach in pharmacy education provided a statistical improvement in student performance, compared to traditional methods. Therefore, flipped classroom is worth promoting in pharmacy education to improve effectiveness and increase student performance. Further research could focus on integrating online and offline education, while instructors could take full advantage of online technology and digital media, and meanwhile construct creative and interactive learning face-to-face atmosphere, contributing to student performance improvement.

### Electronic supplementary material

Below is the link to the electronic supplementary material.

Supplementary Material 1

## Data Availability

The datasets used and/or analysed during the current study available from the corresponding author on reasonable request.

## Abbreviations

CNKI: China National Knowledge Infrastructure
SinoMed: Chinese Biomedical Literature Service System
SMD: The standardized mean difference
CI: Confidence interval
OCSE: Objective Structured Clinical Examination
PRISMA: Preferred Reporting Items for Systematic Reviews and Meta-analysis
EPHPP: Effective Public Health Practice Project
SDs: Standard deviations
PharmD: Doctor of Pharmacy
MCQ: Multiple choice questions
