# Supplementary material for: Effectiveness of flipped classroom in pharmacy education – a meta-analysis
Source: BMC Med Educ. 2023 Nov 17;23:881. doi: 10.1186/s12909-023-04865-2 (PMC10657003; doi:10.1186/s12909-023-04865-2)
Supplement: Supplementary file 1 — Supplementary Material 1 [file 12909_2023_4865_MOESM1_ESM.docx]

SUPPLEMENTARYs

**TableS1** Student Performance of Included Studies

| **Study ID** | **Total** | **CG** | **CG score** | **CG SD** | **IG** | **IG score** | **IG SMD** | **P** |
| --- | --- | --- | --- | --- | --- | --- | --- | --- |
| Prescott, et al. 2016, comp 1 | 258 | 108 | 90 | 4.3 | 123 | 92.3 | 3.1 | p<0.001 |
| Prescott, et al. 2016, comp 2 | 226 | 97 | 85.8 | 4.2 | 129 | 90.3 | 4.9 | p<0.001 |
| He, et al. 2019 | 137 | 56 | 80.05 | 5.59 | 81 | 88.21 | 5.95 | p<0.001 |
| Kangwantas, et al. 2017 | 50 | 21 | 6.19 | 1.76 | 29 | 7.24 | 1.24 | p=0.028 |
| Gloudeman, et al. 2018 | 206 | 104 | 77.8 | 16.8 | 102 | 80.5 | 15.8 | p=0.253 |
| Goh, et al. 2019 | 137 | 74 | 37.23 | 14.74 | 63 | 54.64 | 16.14 | p<0.001 |
| Taglieri, et al. 2017 | 588 | 283 | 90.2 | 7.4 | 305 | 89.1 | 4.4 | p=0.022 |
| Lockman, et al. 2017 | 318 | 156 | 67.01 | 9.6 | 162 | 79.34 | 9 | p<0.0001 |
| Lockman, et al. 2017 | 318 | 156 | 77.23 | 12.43 | 162 | 82.3 | 10.25 | p<0.0001 |
| Koo, et al. 2016 | 178 | 89 | 83.4 | 7.9 | 89 | 88.2 | 7.3 | p<0.001 |
| Munson, et al. 2015 | 238 | 125 | 80.4 | 16.1 | 113 | 82.3 | 14.9 | P=0.06 |
| Wong, et al. 2014, comp.1 | 206 | 105 | 84.1 | 1.9 | 101 | 88.3 | 1.9 | p=0.12 |
| Wong, et al. 2014, comp.2 | 206 | 105 | 56.8 | 2.2 | 101 | 89.6 | 2 | P<0.001 |
| Wong, et al. 2014, comp.3 | 206 | 105 | 73.7 | 2.1 | 101 | 89.2 | 1.4 | P<0.001 |
| Bossaer, et al. 2016 | 148 | 72 | 89.5 | 6.8 | 76 | 88 | 6.9 | p=0.18 |
| Cotta, et al. 2016 | 316 | 165 | 77.6 | 12.7 | 151 | 81 | 11 | p=0.013 |
| McLaughlin, et al. 2014 | 315 | 153 | 160.06 | 14.65 | 162 | 165.48 | 13.34 | p=0.001 |
| McLaughlin, et al. 2013 | 35 | 13 | 154.1 | 16.2 | 22 | 152.1 | 15.6 | p=0.31 |
| Pierce, et al. 2012 | 139 | 68 | 77.7 | 4.7 | 71 | 81.6 | 4.4 | p=0.024 |
| Stewart, et al. 2013 | 136 | 65 | 77.6 | 1.4 | 71 | 72.8 | 1.3 | p=0.013 |
| Donihi, et al. 2014 | 256 | 123 | 80.2 | 9.9 | 133 | 82.8 | 9.4 | p=0.048 |
| Sumanasekera, et al. 2020, comp.1 | 164 | 91 | 91.5 | 2.54 | 73 | 97.6 | 0.93 | p<0.05 |
| Sumanasekera, et al. 2020, comp.2 | 164 | 91 | 85.4 | 2.79 | 73 | 91 | 3 | p<0.05 |
| Sumanasekera, et al. 2020, comp.3 | 164 | 91 | 85.3 | 3.54 | 73 | 91.1 | 2.13 | p<0.05 |
| Sumanasekera, et al. 2020, comp.4 | 164 | 91 | 84.1 | 4.94 | 73 | 92.9 | 2.79 | p<0.05 |
| Nazar, et al. 2019 | 132 | 63 | 84.2 | 6.8 | 69 | 82.2 | 6.3 | p=0.65 |
| Anderson, et al. 2017 | 70 | 32 | 71.3 | 14.7 | 38 | 61.8 | 17.7 | p=0.017 |
| Chen, et al. 2020 | 93 | 44 | 48.14 | 20.58 | 49 | 61.02 | 15.71 | p<0.001 |
| Wang, et al. 2019 | 60 | 30 | 80.13 | 9.58 | 30 | 87.5 | 10.23 | p<0.05 |

*CG=Controlled groups; IG=Intervention groups; comp=comparing groups.*

**TableS2** Search Strategy of Databases

| Database | Search strategy | Matched records |
| --- | --- | --- |
| Cochrane Library | ((flipped classroom) OR (flipped education) OR (flipped learning) OR (reverse classroom) OR (backward classroom) OR (inverted classroom) OR (inverse classroom)) AND (pharmac*) | 112 |
| PubMed | ((flipped classroom) OR (flipped education) OR (flipped learning) OR (reverse classroom) OR (backward classroom) OR (inverted classroom) OR (inverse classroom)) AND (pharmac*) | 122 |
| Embase | ((flipped classroom) OR (flipped education) OR (flipped learning) OR (reverse classroom) OR (backward classroom) OR (inverted classroom) OR (inverse classroom)) AND (pharmac*) | 181 |
| ScienceDirect | ((flipped classroom) OR (flipped education) OR (flipped learning) OR (reverse classroom) OR (backward classroom) OR (inverted classroom) OR (inverse classroom)) AND (pharmac!) | 85 |
| Web of science | (TS=(("flipped classroom" OR "flipped education" OR "flipped learning" OR "reverse classroom" OR "backward classroom" OR "inverted classroom" OR "inverse classroom") AND "pharmac*")) | 16 |
| China National Knowledge Infrastructure (CNKI) | ((flipped classroom) OR (reverse classroom) OR (inverted classroom)) AND (pharmacy education) in Chinese | 35 |
| Chinese Biomedical Literature Service System (SinoMed) | ((flipped classroom) OR (reverse classroom) OR (inverted classroom)) AND (pharmacy education) in Chinese | 291 |


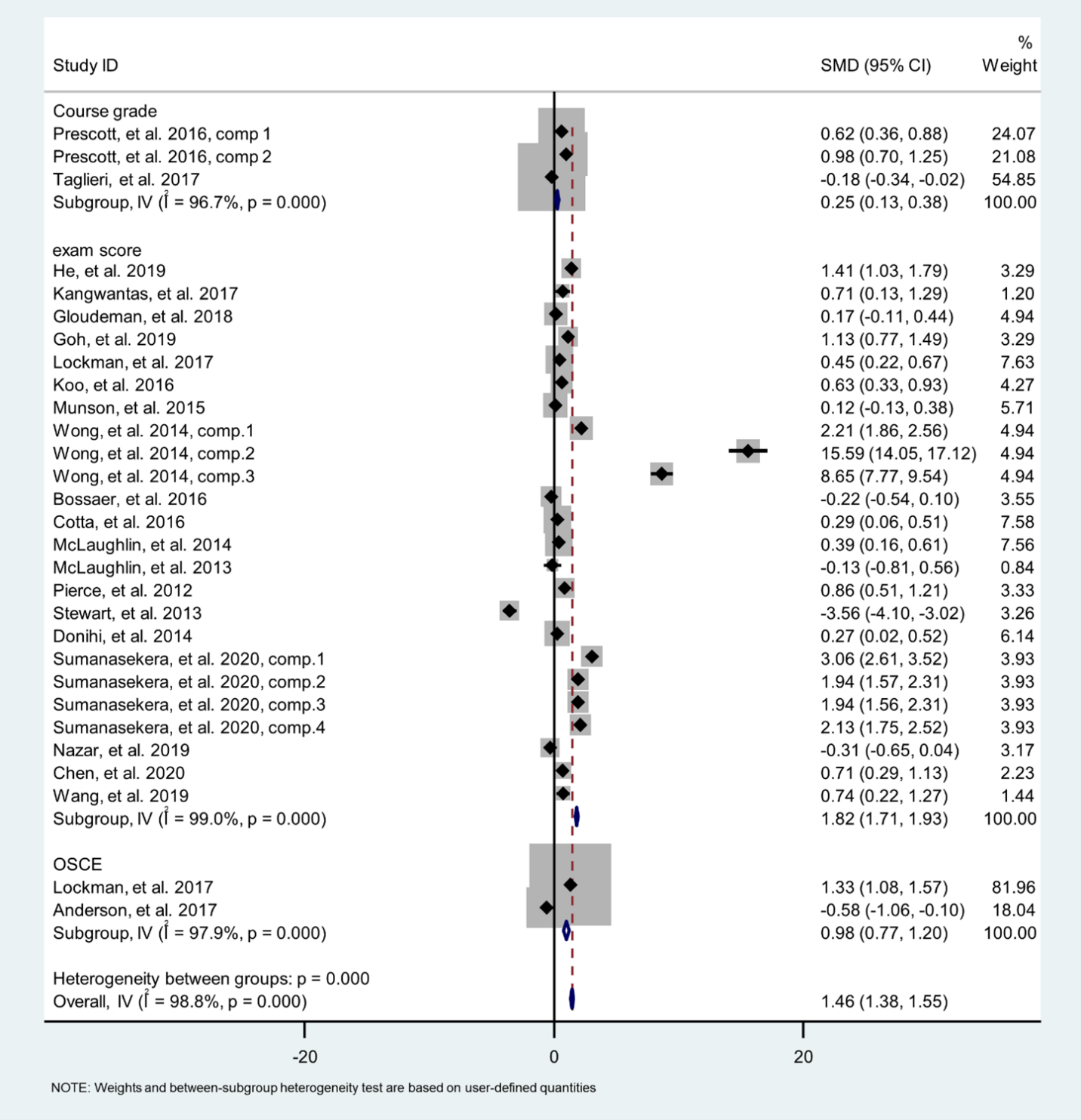


**Fig.S1.** Forest plot for the effectiveness by outcome measures. OSCE= Objective Structured Clinical Examination.


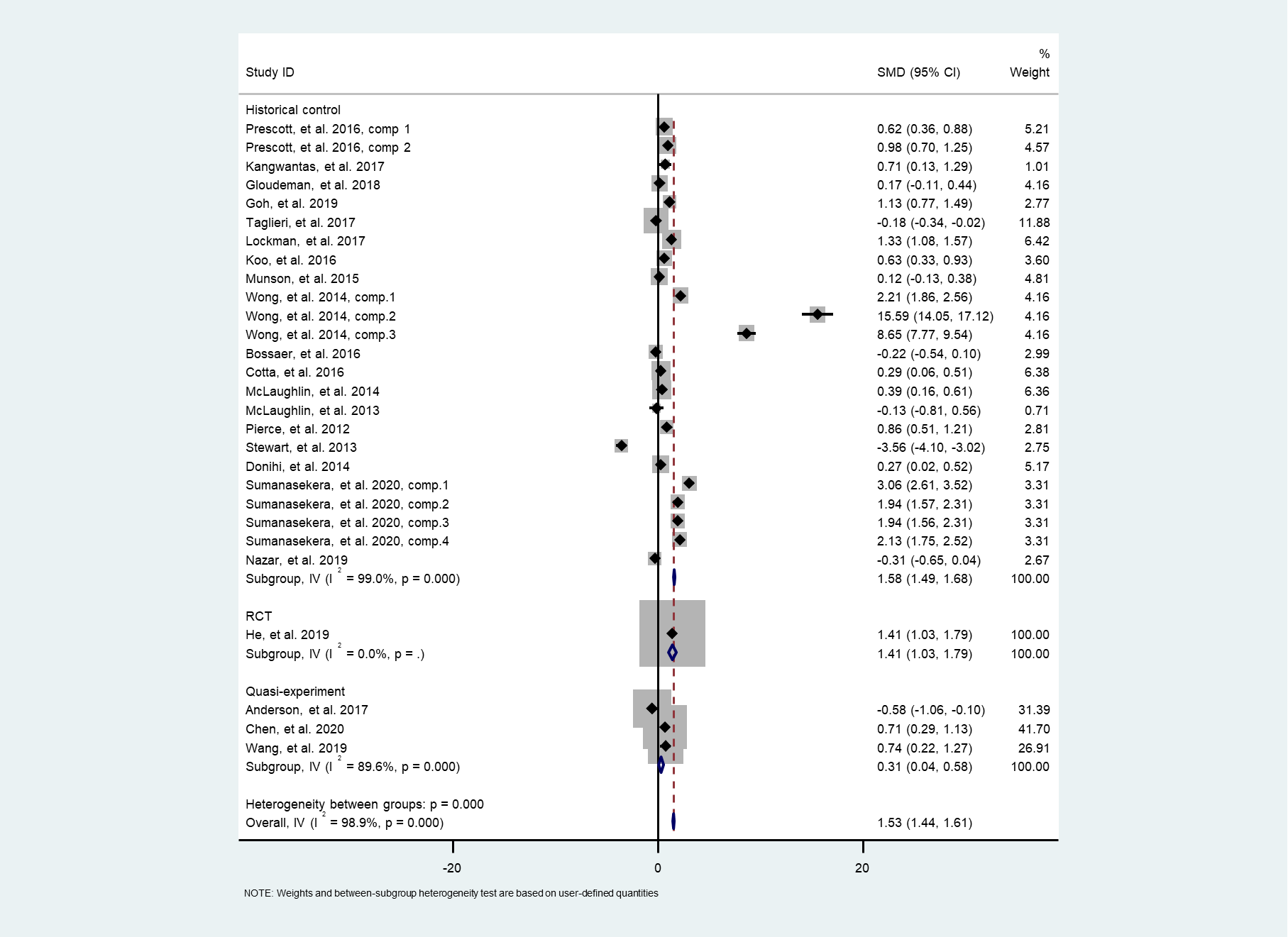


**Fig.S2.** Forest plot for the effectiveness by research design


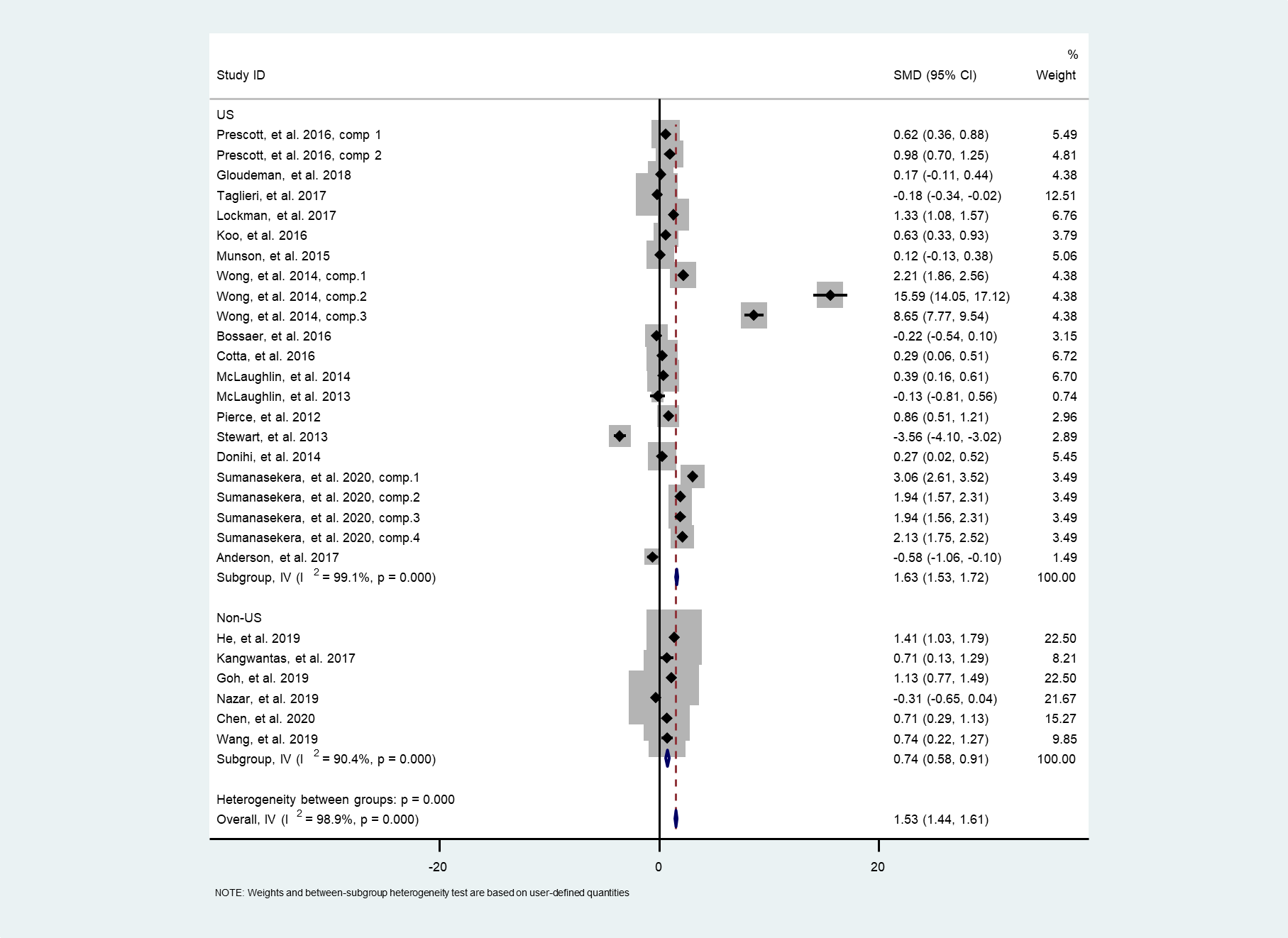


**Fig.S3.** Forest plot for the effectiveness by country of origin


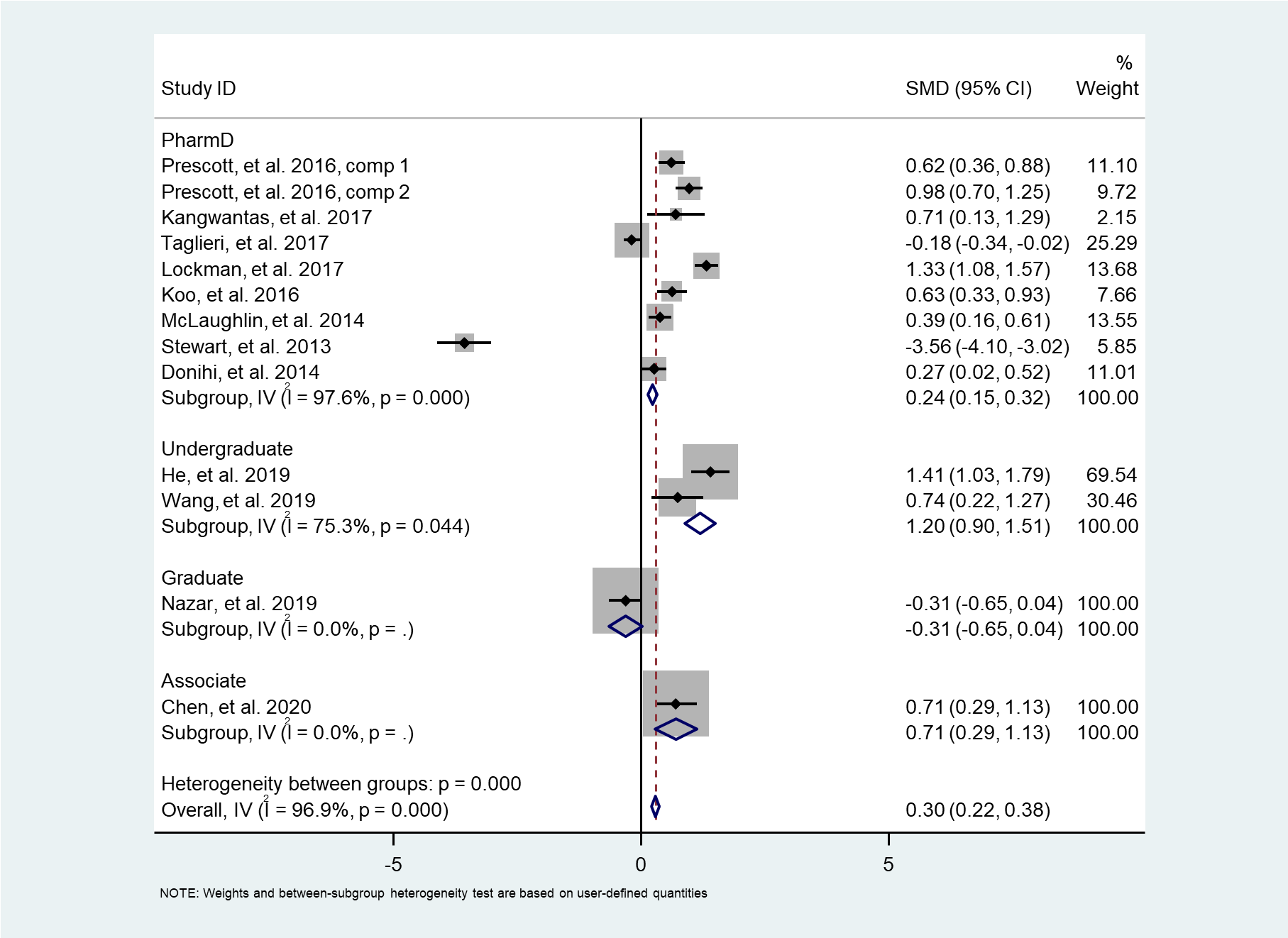


**Fig.S4.** Forest plot for the effectiveness by degree programs


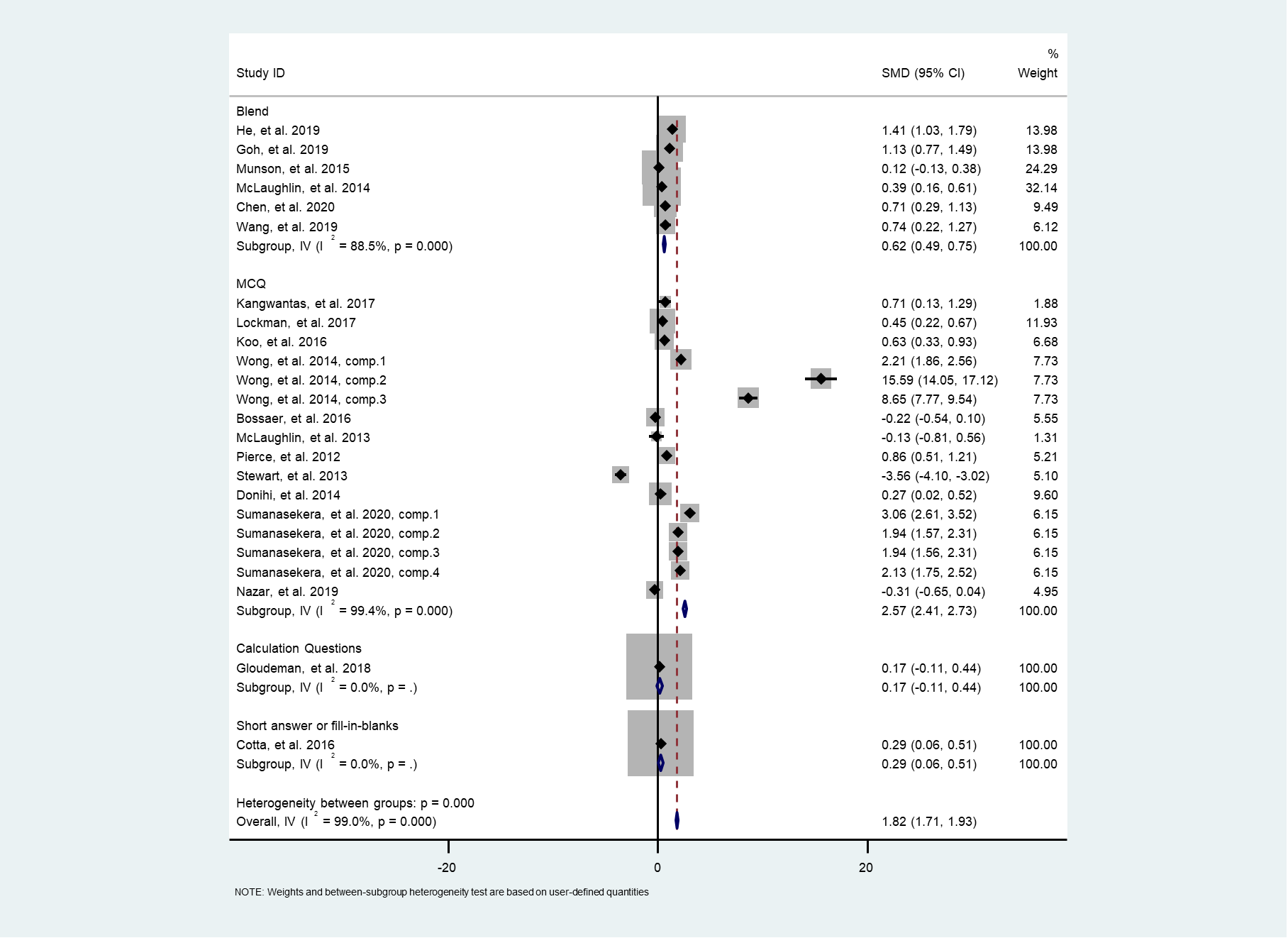


**Fig.S5.** Forest plot for the effectiveness by test format


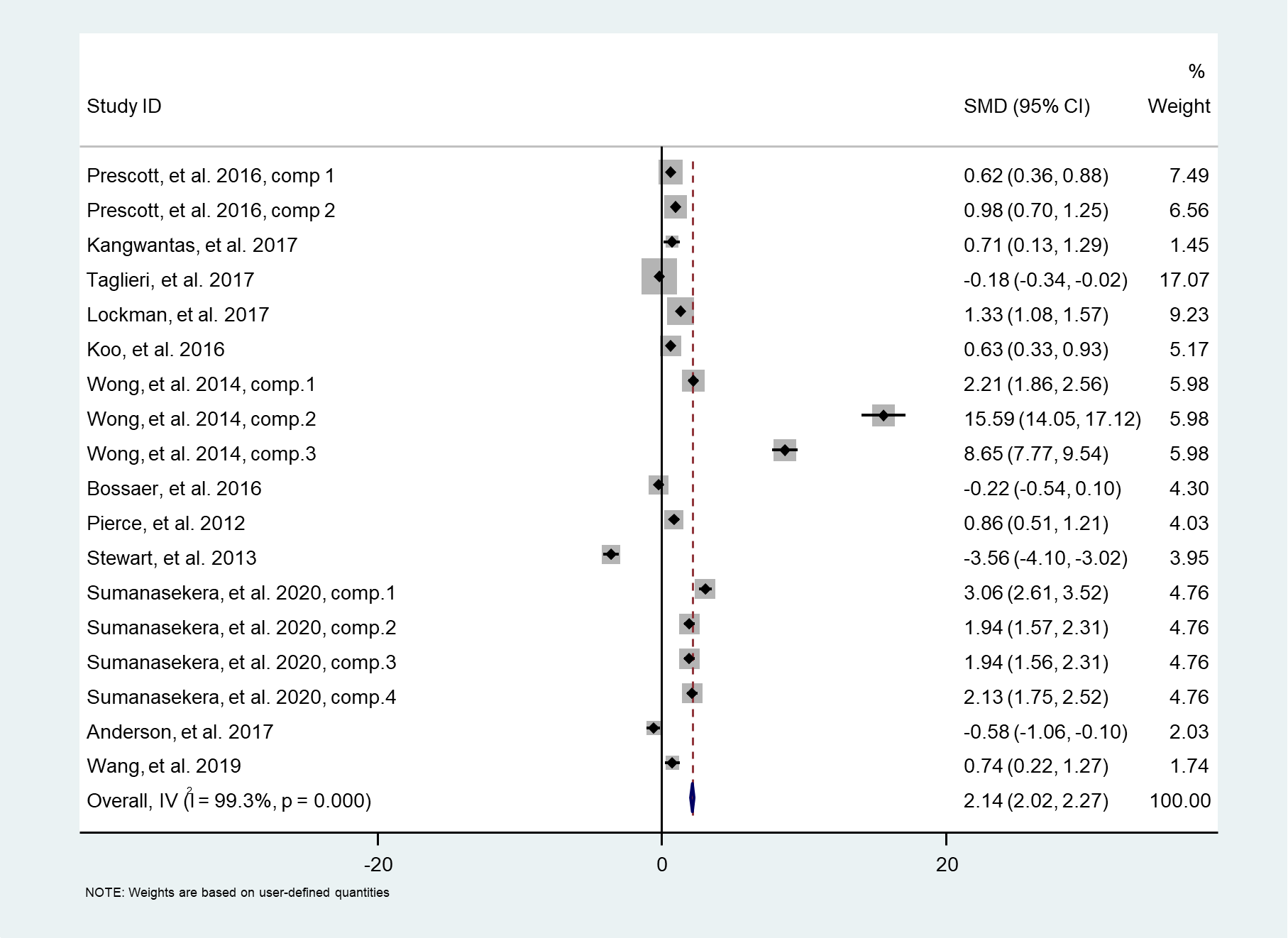


**Fig.S6.** Forest plot for the effectiveness by “incorporating patient case scenario”


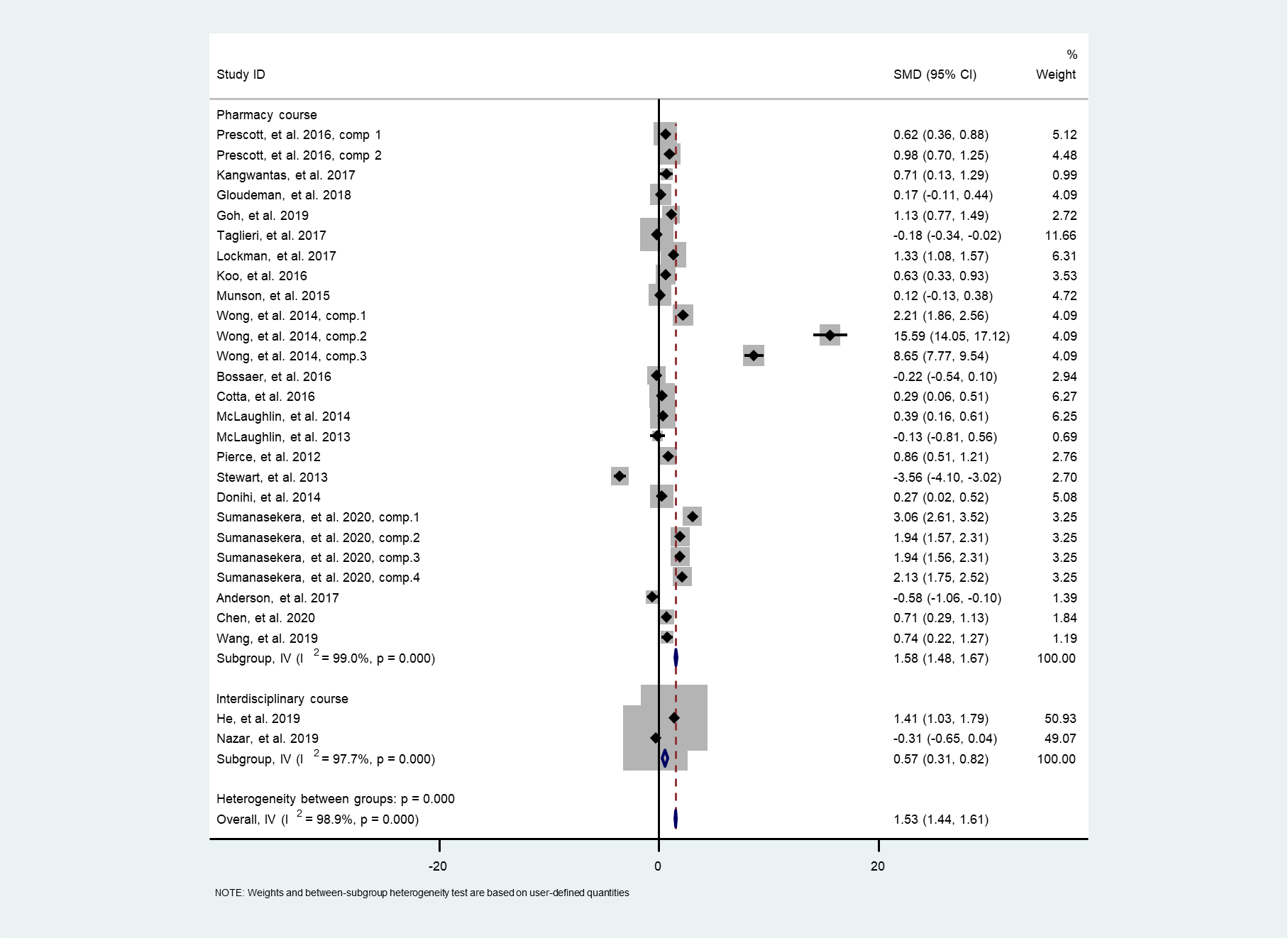


**Fig.S7.** Forest plot for the effectiveness by course type


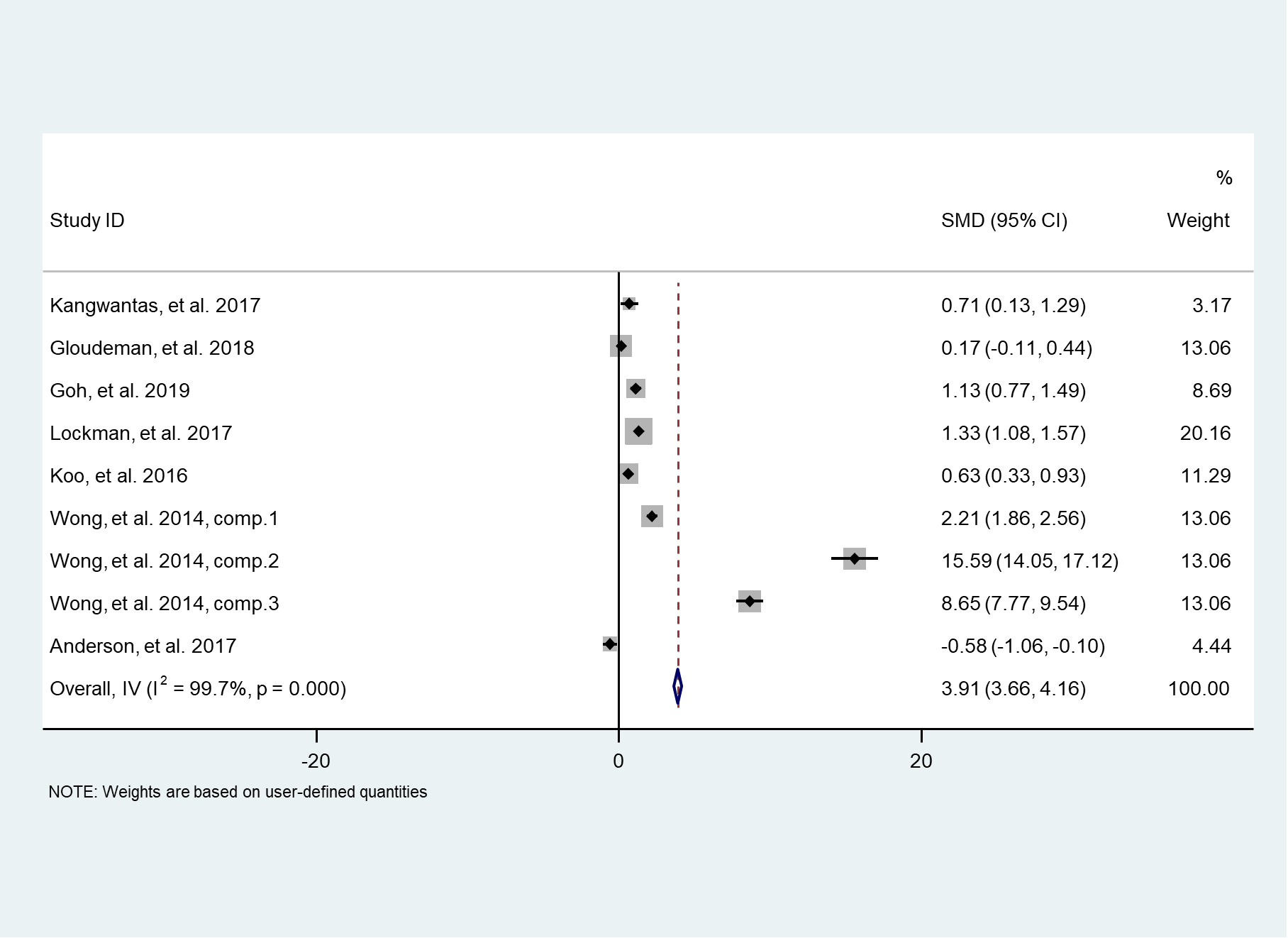


**Fig.S8.** Forest plot for the effectiveness by “availability of quiz at the start of class”


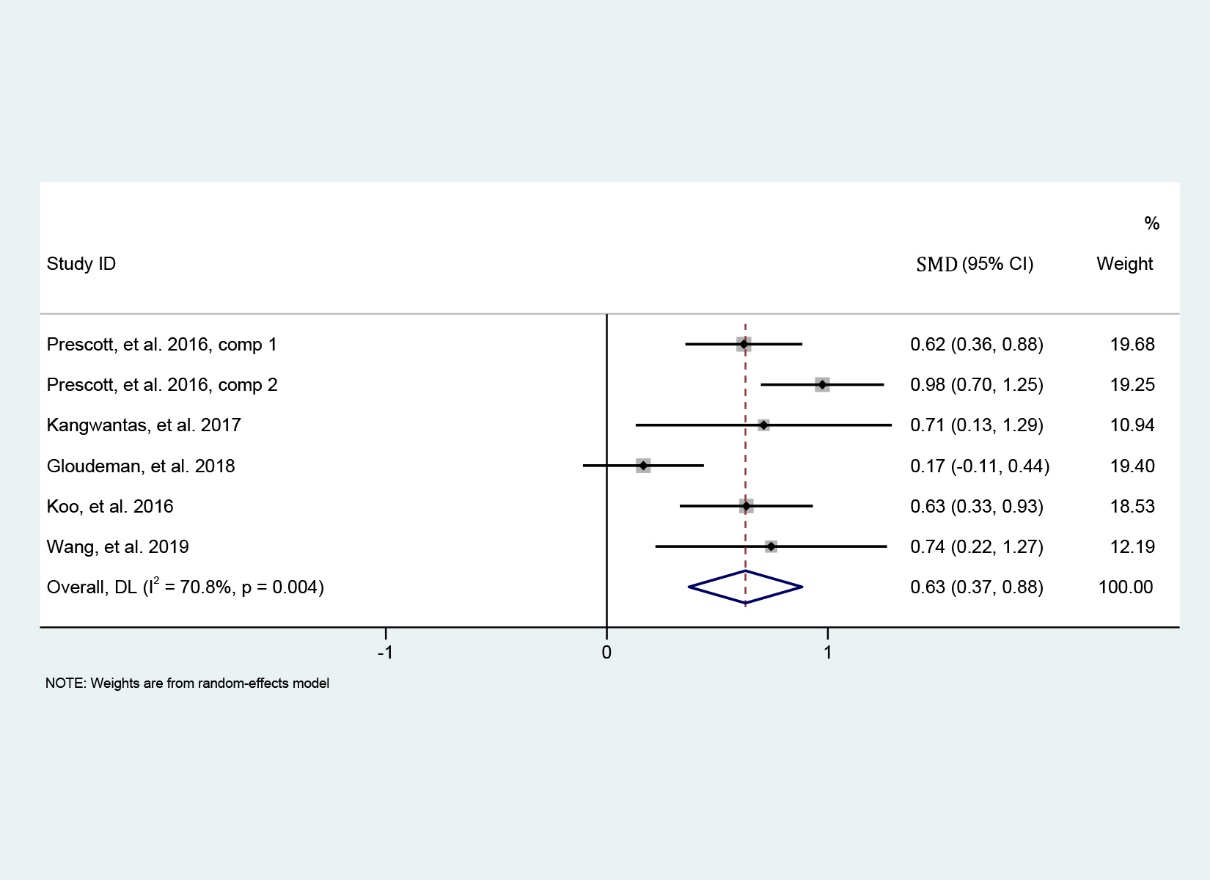


**Fig.S9.** Forest plot for the effectiveness by “pre-class video less than 18 mins”

**Fig.S10.** Funnel plot of all included studies
